# A novel TRPM8 agonist relieves dry eye discomfort

**DOI:** 10.1186/s12886-017-0495-2

**Published:** 2017-06-26

**Authors:** Jee Myung Yang, Fengxian Li, Qin Liu, Marco Rüedi, Edward Tak Wei, Michael Lentsman, Hyo Seok Lee, Won Choi, Seong Jin Kim, Kyung Chul Yoon

**Affiliations:** 1 Graduate School of Medical Science and Engineering, Korea Advanced Institute of Science and Technology, Daejeon, South Korea; 2 Department of Ophthalmology, Chonnam National University Medical School and Hospital, 42 Jebong-ro, Dong-gu, Gwangju, 61469 South Korea; 3 Department of Anesthesiology, Zhujiang Hospital, Southern Medical University, Guangzhou, China; 4 Department of Anesthesiology and Center for the Study of Itch, Washington University, School of Medicine, St. Louis, MO USA; 5 Alveonix AG, Rotkreuz-Zug, Switzerland; 6 Alta Research LLC, Berkeley, CA USA; 7 Pavlov Institute of Physiology, 199034 St. Petersburg, Russia; 8 Department of Dermatology, Chonnam National University Medical School and Hospital, 42 Jebong-ro, Dong-gu, Gwangju, 61469 South Korea

**Keywords:** Dry eye, Eyelid, Ocular discomfort, TRPM8

## Abstract

**Background:**

Physical cooling of the eye surface relieves ocular discomfort, but translating this event to drug treatment of dry eye discomfort not been studied. Here, we synthesized a water-soluble TRPM8 receptor agonist called cryosim-3 (C3, 1-diisopropylphosphorylnonane) which selectively activates TRPM8 (linked to cooling) but not TRPV1 or TRPA1 (linked to nociception) and tested C3 in subjects with mild forms of dry eye disease.

**Methods:**

A set of 1-dialkylphosphoryalkanes were tested for activation of TRPM8, TRPV1 and TRPA1 receptors in transfected cells. The bioactivity profiles were compared by perioral, topical, and intravenous delivery to anesthetized rats. The selected lead candidate C3 or vehicle (water) was applied with a cotton gauze pad to upper eyelids of patients with dry eye disease (*n* = 30). Cooling sensation, tear film break-up time (TBUT), basal tear secretion, and corneal staining were evaluated. C3 was then applied four times daily for 2 weeks to patients using a pre-loaded single unit applicator containing 2 mg/mL of C3 in water (*n* = 20) or water only. TBUT, basal tear secretion, and corneal staining, and three questionnaires surveys of ocular discomfort (VAS scale, OSDI, and CVS symptoms) were analyzed before and at 1 and 2 weeks thereafter.

**Results:**

C3 was a selective and potent TRPM8 agonist without TRPV1 or TRPA1 activity. In test animals, the absence of shaking behavior after C3 perioral administration made it the first choice for further study. C3 increased tear secretion in an animal model of dry eye disease and did not irritate when wiped on eyes of volunteers. C3 singly applied (2 mg/ml) produced significant cooling in <5 min, an effecting lasting 46 min with an increase in tear secretion for 60 min. C3 applied for 2 weeks also significantly increased basal tear secretion with questionnaire surveys of ocular discomfort indices clearly showing improvement of symptoms at 1 and 2 weeks. No complaints of irritation or pain were reported by any subject.

**Conclusions:**

C3 is a promising candidate for study of TRPM8 function on the eye surface and for relief of dry eye discomfort.

**Trial registration:**

ISRCTN24802609 and ISRCTN13359367. Registered 23 March 2015 and 2 September 2015.

**Electronic supplementary material:**

The online version of this article (doi:10.1186/s12886-017-0495-2) contains supplementary material, which is available to authorized users.

## Background

The ocular surface, comprising the epithelia of the cornea, limbus, conjunctiva, and eyelid margins, is vulnerable to dysfunction because of constant exposure to the external environment. The limited repertoire of self-defense, of eyelid closure and the tear film, may not be sufficient to cope with injury. Desiccation is a major threat to the integrity of the ocular surface and is caused by inadequate tear secretion, poor tear quality, inflammation of ocular surface or reduced frequency of blinking [1, 2]. Dry eye is a symptom of the computer vision syndrome (CVS, or digital eye strain) when one stares, for example, at a video screen for >3 h [3]. Dry eye, as a disease (DED), affects 5–30% of the population and is an economic burden to society [4–7]. Recent improvements in the knowledge of pathophysiology of DED enable strategic approaches in the treatment of DED, and emerging drugs are targeted to efficiently reduce the patients’ discomfort [8, 9]. The dense neural network of the ocular surface, especially of the cornea, generates the signs and symptoms of DED, namely, redness and tearing, and irritation, itch, pain and dysesthesia such as feelings of grittiness, soreness, the presence of a foreign object, dryness, and eye fatigue [10–12]. The coding of neural circuits of the ocular surface is a subject of intense research [13–16]. For example, the distributions of transient receptor potential vanilloid 1 (TRPV1), and transient receptor potential melastatin 8 (TRPM8) ion channels on the cornea have been mapped, and it is likely that TRPV1 transduces the signals of heat, irritation, and pain from the ocular surface [15, 17]. The role of TRPM8 is multifaceted. TRPM8 may be associated with the detection of “dryness” on the eye surface because it is activated by evaporative cooling and by hyperosmolar solutions [18, 19]. TRPM8 may also be a direct stimulator of tear secretion from the lacrimal gland [20]. So far, translation of these research findings to therapy of dry eye has not been clearly defined for TRP drug targets, or for studies of lead candidates, animal models of disease, mechanisms of action, or clinical observations [11, 21].

**Fig. 1:**
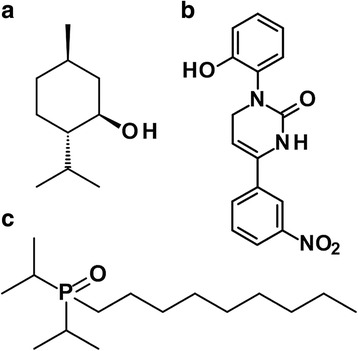
Structure of I-menthol (**a**), icilin (**b**) and C3 (**c**)

TRPM8 is the principal receptor protein of cold-sensitive nerve fibers associated with the detection of cooling sensations on body surfaces such as the skin [17, 22]. But it is less clear how a TRPM8 agonist applied to the ocular surface will affect sensation or discomfort. Experience has shown that an ice pack applied to the orbit reduces the pain of injury [23]. In studies on humans, cooling relieves the pain of cataract surgery and artificial tears kept at 4 °C elevate the threshold pressure for detecting a microfilament applied to the eye surface, suggesting that TRPM8 activation is beneficial for discomfort [23, 24]. But the utility of cooling for the dysesthesia of DED is uncertain. DED patients display a corneal hypersensitivity to normally innocuous cold stimuli (cold allodynia) [25]. Standard TRPM8 agonists such as menthol and icilin (Fig. 1a, b) are not fit for ocular studies. Menthol vapors irritate the eye and menthol solutions causes stinging followed by a brief episode of cooling [26]. Icilin, a more potent TRPM8 agonist than menthol, was reported to produce punctate and long-lasting cooling on the eyelids, but this information is anecdotal [27]. Icilin is difficult to study because it is not soluble in any ophthalmic vehicles and thus difficult to formulate for delivery [28]. Antagonism of TRPM8 has also been considered for DED because evaporative cooling and hyperosmotic stimuli may trigger dry eye pain [11, 19]. But antagonists may reduce tear secretion and this would be an undesirable side effect [20]. No ocular symptoms were described when 22 volunteers were given an experimental TRPM8 antagonist [29].

Here, the strategy was to apply a water-soluble TRPM8 agonist onto the upper eyelid margins with the goal of reducing eye discomfort from dryness and from DED. The topical delivery to the eyelid margins was achieved with a cotton wipe or swab saturated with drug solution. Eye drops are the most common form of ocular drug delivery but drops can exacerbate discomfort when drug molecules contact the cornea, a surface densely innervated with nociceptors and super-sensitive to painful stimuli. The use of wipe or a cotton-tipped applicator minimizes drug contact with the cornea which occupies approximately 1/6 of the total area of the anterior eyeball [30]. The stimulation of TRPM8 receptors on the eyelids is designed to impart a cooling, refreshing, and energizing sensations to the brain, with avoidance of sting, irritation or pain [31, 32]. In choosing a lead candidate for study, the desired qualities of the molecule are: potency in TRPM8 receptor assays, selective activity on TRPM8 and not on nociceptors such as TRPV1 or TRPA1, aqueous solubility to facilitate formulation and delivery, a duration of drug action compatible with clinical use, positive activity in a meaningful animal model of injury, a clear defined mechanism of action, and the absence of, or reduced irritant action or side effects, when applied to the eyes of humans. A class of chemicals called dialkylphosphorylalkanes [33] was examined because they are soluble in water at effective concentrations of 0.1 to 5 mg/mL. A lead candidate called cryosim-3, abbreviated as C3, (1-diisopropylphosphorylnonane, CAS Registry Number 1503744–37–8-7) was identified as having the desirable characteristics of a non-irritating selective TRPM8 agonist (Fig. 1c) [34]. C3 is active in a mouse model of DED and relieves ocular discomfort in subjects diagnosed with DED in our clinic. C3 is an ideal reagent for further study of the sensory discomfort caused by a dry eye.

## Methods

### Chemical synthesis

The compounds tested here are trialkyl derivatives of phosphoric acid. (dialkylphosphorylalkanes or Dapa), in which two of the alkyls are either isopropyl or sec-butyl, and the third alkyl is C_**4**_ to C_9_ (Additional file 1: Table S1). The Dapa were custom synthesized by Dr. J.K. Chang of Phoenix Pharmaceuticals, Inc. (Burlingame, CA), using this general method: 100 mL (23.7 g, ~200 mmol) of isopropylmagnesium chloride or sec-butylmagnesium chloride were obtained from Acros, as a 25% solution in tetrahydrofuran (THF) and placed under nitrogen in a 500 mL flask (with a stir bar). Diethylphosphite solution in THF (from Aldrich, D99234; 8.25 g, 60.6 mmol in 50 mL) was added drop-wise. After approximately 30 min, the reaction mixture warmed up to boiling. The reaction mixture was stirred for an extra 30 min, followed by a drop-wise addition of the appropriate *n*-C_**4**_ to C_**9**_ iodide solution in THF (from TCI; 60 mmol in 20 mL). The reactive mixture was then stirred overnight at room temperature. The reaction mixture was diluted with water, transferred to a separatory funnel, acidified with acetic acid (~10 mL), and extracted twice with ether. The ether layer was washed with water and evaporated (RotaVapBuchi, bath temperature 40 °C). The light brown oil was distilled under high vacuum (0.5 mmHg). The final products, mass verified by mass spectrometry, were transparent liquids that were colorless or slightly pale yellow and have boiling points in the range of 120 to 130 °C. Several samples of 1-diisopropylphosphorylheptane and 1-diisopropylphosphorylnonane were sent for analysis by gas chromatography-mass spectrometry (GC-MS, NDE Analytical, Pleasanton, California, USA, http://www.ndeanalytical.com) on an Agilent GC/MS system 6890/5973 equipped with a TraceGold TG-624 column, with helium as the carrier gas (flow rate: 1.6 mL/min) and the injector port set at 220 °C (split ratio 50:1, temperature program: 100 to 240 °C). The main components of the total ion chromatogram (TIC) had a retention time of 13 to 14 min, and 18 to 19 min, and the detected peaks accounted for 98.7 and 97.2% of total area, for 1-diisopropylphosphorylnonane and 1-diisopropylphosphorylheptane, respectively.

### TRPM8, TRPA1, and TRPV1 receptor assays

Compounds were tested on Chinese Hamster Ovary (CHO) cells stably transfected with human TRPM8 cDNAs using a Fluo-8 calcium kit and a Fluorescence Imaging Plate Reader (FLIPRTETRA™) instrument. Assays were conducted by ChanTest Corporation, 14,656 Neo Parkway, Cleveland, OH 44128, USA. Solutions were prepared by diluting stock solutions in a HEPES-buffered physiological saline (HBPS) solution. Test compound and control formulations were loaded in polypropylene or glass-lined 384-well plates, and placed into the FLIPR instrument (Molecular Devices Corporation, Union City, CA, USA). Each was tested at 8 concentrations with *n* = 4 replicates per determination. The positive control reference compound was l-menthol, a known TRPM8 agonist. For FLIPRTETRA™ assay, cells were plated in 384-well black wall, flat clear-bottom microtiter plates (Type: BD Biocoat Poly-D-Lysine Multiwell Cell Culture Plate) at approximately 30,000 cells per well. Cells were incubated at 37 °C overnight to reach a near confluent monolayer appropriate for use in a fluorescence assay. The test procedure was to remove the growth media and to add 40 μL of HBPS containing Fluo-8 for 30 min at 37 °C. 10 μL of test compound, vehicle, or control solutions in HBPS were added to each well and read for 4 min. Concentration-response data were analyzed via the FLIPR Control software that is supplied with the FLIPR System (MDS-AT) and fitted to a Hill equation. The 12 compounds tested showed full efficacy on the TRPM8 receptor, i.e., at higher tested concentrations there was ~100% stimulation of calcium entry, and the data fitted a sigmoidal dose-response curve.

To further examine the specificity of C3, tests were conducted on TRPV1 channels and TRPA1 channels expressed in Kirsten murine sarcoma virus transformed rat kidney (KNRK) cells. KNRK cells were cultured as a monolayer and maintained in Dulbeccos’s Modified Eagles’s Medium (Life Technologies), supplemented with 10% fetal bovine serum (Life Technologies), 100 units/mL penicillin and 100 μg/mL streptomycin, in an incubator of 5% CO_2_ at 37 °C. After suspension, the cells were coated on cover slips for 12 h, then transiently transfected with cDNA (pc3.1 DNA) for TRPV1 or TRPA1 with Lipofectamine 2000 (Invitrogen) for 24 h, and loaded with Fura-2 AM™ (Molecular Probes) for 40 min at 37 °C. After washing and recovery, KNRK cells were imaged at 340 and 380 nm excitation to detect free calcium influx. An increase of 50% of the 340/380 ratio was considered as the response threshold which were measured under masked conditions for plasmids. Compounds were applied to the bath and calcium response was acquired by an inverted Nikon fluorescence microscope with a CoolSnap HQ2 CCD camera (Photometrics, Tucson, AZ). Data were quantified offline with the Nikon-NIS program.

### Shaking activity after intravenous, perioral, and topical administration

Male rats weighing 220–260 g were anesthetized with sodium pentobarbital, 55 mg/kg intraperitoneal, and after the loss of the righting reflex, animals were placed on a table and body temperature was recorded. The femoral vein was cannulated with polyethylene-20 tubing connected to a 1 mL syringe. For the intravenous and perioral routes, 0.1 mL of solution was given per 100 g body weight at a dose of 2 mg/kg or 20 mg/kg, respectively. For topical administration, the abdominal skin was shaved and 20 μL of the pure Dapa was applied with a micropipette on a ~ 1 cm diameter circle of skin, enclosed with a ring of cream (Baby cream “NevskayakosmetikaDetskyi” NevskayaKosmetika Inc., Saint-Petersburg 192,029). Animals were observed and shaking frequency counted for 15, 40 min and 1 h after intravenous, perioral, and topical applications, respectively. There were *n* = 3 to 6 rats per test substance. For the intravenous route, two trials were conducted in the same animal with a 10 to 15 min interval between doses. The shaking frequency shown in the graphs is for the second trial. Shaking behavior are rapid alternating contractions of the supination and pronation muscles about the spinal axis, and can be readily observed in the readily observed in the unanesthetized or anesthetized state and counted (Additional file 2: Movie S1). The pattern of response after intravenous, perioral, and topical delivery provides information on the ability of the molecule to cross membrane barriers. Experiments on rats were ended by euthanasia with intraperitoneal injection of sodium pentobarbital 150 mg/kg.

### Primary sensory neuron studies in mouse

Calcium imaging was used to test the C3 selectivity on TRPM8-sensory neurons. *Trpm8* ^*+/EGFP*^mice were gifted by Dr. Yu-Qing Cao, Pain Center of Washington University in Saint Louis, School of Medicine, Missouri. Trigeminal ganglions and dorsal root ganglions from 4 to 5 weeks old mice were collected after CO_2_ euthanasia and digested for 30–40 min before plated on cover slips (8 mm), which were coated with poly-D-lysine for 40 min before use. Cover slips were gently washed with culture media to remove myelin after incubating for 35–40 min. Calcium imaging was done after incubation overnight at 37 °C, 5% CO_2_. Neurons were loaded with Fura-2 AM™ (Molecular Probes) for 30 min at room temperature. After washing and recovery, neurons were imaged at 340 and 380 nm excitation to detect free calcium influx. An increase of 50% of the 340/380 ratio was considered as the response threshold which were measured under blinded conditions for genotypes. Compounds were applied to the bath and calcium response was acquired by an inverted Nikon fluorescence microscope with a CoolSnap HQ2 CCD camera (Photometrics, Tucson, AZ). Data was quantified offline with the Nikon-NIS program.

In the retrograde labeling and immunofluorescence study, *Trpm8* ^*EGFPf/+*^transgenic mice were anesthetized with xylazine (3 mg/kg) and ketamine (15 mg/kg) mixture for dye injection and perfusion. Neuronal tracer Fluoro-Gold™ (2 μl, 4%, dissolved in distilled H_2_O) was injected into the upper eyelid skin with a needle made from a fine glass capillary tube. Mice were perfused with 4% paraformaldehyde in PBS (pH 7.2, 4 °C), followed by PBS (pH 7.2, room temperature) on the 5th day after dye injection. Trigeminal ganglions were collected and frozen in 20% sucrose overnight and then sectioned at 12 μm onto slides for staining. Whole-mounts of upper eyelid skin were collected from non-dye-injection *Trpm8* ^*EGFPf/+*^ mouse and post-fixed with 4% paraformaldehyde on ice for 2 h after CO_2_ euthanasia, then washed with PBS for 3 times before immunostaining. Immunofluorescence staining was done as described previously [35]. Slides and upper eyelid skin were washed with PBS in 0.2% Triton X-100 (PBST) for 3 times and blocked with 10% donkey serum in phosphate buffered saline with Tween 20 for 2 h, then were incubated in chicken anti-GFP (GFP-1020; Aves Lab; 1:1000) solution at 4 °C for 24 h. Donkey anti-chicken IgG (114,050, FITC conjugated;Jackson ImmunoResearch; 1:1000) was incubated for 2 h at room temperature after 3 times washes with PBST. Sections and whole mount of eyelid skin, cornea, and dissected conjunctiva were washed with PBS and mounted with Fluoromount-G (Southern Biotech). Images were taken and analyzed using Nikon fluorescence microscope with a CoolSnap HQ2 CCD camera (Photometrics, Tucson, AZ).

In the double retrograde labeling experiment (Additional file 1: Figure S2), *wild-type* mice were anesthetized with xylazine (3 mg/kg) and ketamine (15 mg/kg) cocktail for dye injection and perfusion. Neuronal tracer, the fluorescence-conjugated-wheat germ agglutinin (WGA, Invitrogen, Molecular Probes, Inc. Eugene, OR 97402, 5 mg/mL, dissolved in PBS), was injected into the upper eyelid skin (1 μL, WGA-Alexa Fluor® 555, Lot# W32464) and the ipsilateral cornea (0.5 μL, WGA-Alexa Fluor® 488, Lot# W11261) with a fine glass capillary. Trigeminal ganglions were collected from 4% paraformaldehyde perfused mice on day 5 after dye injection and cryoprotected overnight before freezing and sectioning sectioned at 12 μm onto slides. Visible fluorescence can be detected directly in the labeled neurons under a Nikon fluorescence microscope.

### Mouse dry eye model

*C57BL/6 J wild-type* mice were purchased from Jackson Laboratory (Stock No. 000664). Adult male mice were anesthetized with xylazine (3 mg/g) and ketamine (15 mg/g) mixture and incisions of 5 mm were made in the skin between the eye and the ear, both extraorbital lacrimal glands were gently isolated by forceps and removed [36]. As mouse has three pairs of lacrimal glands, removing the extraorbital ones will induce partially tear secretion deficiency, but still have other tear sources to be triggered [37]. Sham mice received the same procedure without gland removal. Skin was sutured with 6–0 black monofilament nylon (Ethilon from Ethicon, Inc.). All mice received antibiotics (100 μL Enroflox™, intramuscular daily) and topical analgesia (2% lidocaine gel) for 2 days postoperative. Behavioral assays were done between 2 and 4 weeks after the surgery.

Tear volume was measured with phenol-red cotton threads (Zone-Quick; Showa Yakuhin Kako CO., Ltd., Tokyo, Japan) as described [38]. The threads were held with forceps and applied to the lateral canthus for 30 s. Immediately afterwards the wetting of the thread was read in mm under a dissection microscope. Corneal abrasion was assessed under cobalt blue light after application of 0.5 μl of 0.25% fluorescein sodium (Bausch & Lomb Inc. Tampa, FL 33637). Grades of abrasion were classified with a grading system that is based on area of corneal staining [39]. Grouping was blinded to the observers. Results were grouped according to the treatments after analysis.

### Test in human subjects

This randomized prospective double-masked study was conducted in accordance with the Declaration of Helsinki. Written Informed consent was obtained from all subjects. The clinical trial was registered and assigned an International Standard Randomized Controlled Trial Number (ISRCTN 24802609 and ISRCTN13359367).

A sample size was calculated using the G*Power software (version 3.0.10; Universität Kiel Dusseldorf, Germany) taking into account the results of the pilot study in which the standard deviation of Schirmer score between two groups was 2.2 mm. The sample size required to achieve a level of α = 0.05 and a power of 80% to detect 2.0 mm/5 min difference in basal tear secretion between groups was estimated at 20 or more patients per group. Initially 70 patients were screened for eligibility. Seven patients were excluded because they did not meet the inclusion criteria, and three declined to participate. Sixty healthy young subjects (aged ≥18 years) with mild to moderate DED (Dry Eye Workshop dry eye severity level ≤ 2) [40], first diagnosed at the Ocular Surface Center, Department of Ophthalmology, Chonnam National University Hospital were enrolled.

Inclusion criteria were dry eye symptoms for more than 3 month despite the use of artificial tear, low tear break-up time (TBUT) (≤7 s), low basal tear secretion (≤10 mm/5 min), and presence of corneal and conjunctival epithelial damage [41]. Exclusion criteria included a history of any ocular disease other than DED, contact lens use, ocular trauma or surgeries, and the presence of systemic disease that could affect ocular surface condition. To exclude evaporative type DED, patients who presented two or more morphologic features of the meibomian gland duct orifice and acini on the posterior lid margin, including vascular dilation, acinar atrophy, orifice plugging or metaplasia [42, 43]. Also excluded were subjects with punctual plugs, used eye drops other than artificial tears, used any systemic medication that can cause DED, or who were pregnant. The design of the experiment and the subjects are described (Additional file 1: Figs. S3, S4 and Tables S2, S3).

In the first experiment in which only a single application was tested, 60 subjects were randomly assigned using online software (http://www.graphpad.com/quickcalcs/index.cfm). One group received the vehicle solution (distilled water) (*N* = 30), and the second group received the TRPM8 agonist C3 dissolved in 2 mg/mL in distilled water (*N* = 30). Test solutions were kept at room temperature and topically applied to the eyelid skin and margins using an absorbent cotton gauze square (0.4 g rectangle (50 mm × 60 mm), CS-being, Daisan Cotton, Japan). A loading volume of 0.5 mL of solution was used to wet the cotton and the square wiped twice across the closed eyelids (Fig. 5c). The off-loaded volume from the gauze was estimated by weighing the square before and after wiping and found to be 6.5 mg or 6.5 μL. For the C3 solution this is equivalent to 13 μg of C3 for both eyes. This method of delivery targets the edges of the closed eyelids and utilizes the eyelashes as a wick to distribute the solution to the mucocutaneous junctions, conjunctiva, and precorneal film. The lid wiper mechanism of the blink further evenly distributes the solution on to the ocular surface [44]. By using this wiping method, bolus delivery of an eye drop to the corneal surface is avoided.

Subsequently, cooling sensation (every 5 min), TBUT (every 10 min), basal tear secretion (every 20 min), and keratoepitheliopathy (KEP) score (every 30 min) were evaluated for 1 h. In addition, a questionnaire asking the severity of dry eye was used to evaluate the changes in dry eye symptom before and 1 h after application of topical agents. To avoid the effect of topical anesthesia on patient’s sensation, changes in cooling sensation and symptoms of dry eye were evaluated in the right eye, and the remaining examinations including the basal tear secretion measured by Schirmer score with application of topical 0.5% proparacine were performed on the left eye. All examinations were performed by a single masked investigator (K.C.Y) who was not informed of the results of the randomization. All the experiments and the answering the questionnaire were performed in a separate room so that the subjects would not compare the difference of the medications. The room temperature of the study was maintained at 25 °C. Cooling sensation was recorded by subjects using VAS symptom intensity scores on a scale of 0 (no symptom) to 10 (maximum intensity). The severity of dry eye symptoms was graded on a numerical score of 0 to 4 as follows; 0, no symptoms; 1, mild symptoms; 2, moderate symptoms; 3, severe symptoms; and 4, very severe symptoms [45]. TBUT and basal tear secretion (Schirmer score with application of topical 0.5% proparacaine) were measured as previously described [46]. To assess the corneal epithelial damage, the cornea was stained with 1% Fluorescein dye and the severity of KEP was scored by multiplying the area score (0–3) by density score (0–3) of staining [47].

In the second experiment, to account for potential dropout rate of 25%, the required sample size was 54 subjects, 27 per group. Initially, a total of 60 additional patients fulfilling the inclusion criteria were assessed for eligibility, and 54 subjects who decided to participate were randomized into two groups as described above (Additional file 1: Figure S4). Vehicle or C3 were prepared as 1 mL solutions placed into unit applicators called Swabdose™ made by Unicep Corporation, Sandpoint, ID. These applicators are singly encased in a polypropylene container with a 6.35 mm cotton tip attached to a 74.6 mm polystyrene handle. USP purified water (placebo units) or a C3 sample (2 mg/mL in USP purified water) was prepared onsite under clean conditions and packaged with automated equipment. Each subject was given 14 Swabdoses for the 2-week study period. The subjects were instructed to apply the cotton-tipped applicator to the edges of the closed eyelids once every 6 h per day for 14 days. The applicators were pre-weighed and the subjects were told to use to same applicator for each day and to save the applicator in a plastic bag at the end of the day. The applicators were collected, weighed and compared with the weight before application. TBUT, basal tear secretion, and corneal staining assessed by KEP scores were analyzed before and at 1 and 2 weeks thereafter. For TBUT, basal tear secretion, and KEP only the results from the right eye were scored for data analysis.

Three questionnaires were used at each patient visit to analyze the effects of treatment on ocular discomfort. Firstly, DED symptoms were assessed using a visual analogue scale (VAS) score of 1 to 10. Next, symptoms were assessed using the ocular surface disease index (OSDI) questionnaire, consisting of 12 questions graded on a scale from 0 to 4 (0 = “none of the time” and 4 = “all the time”) [48]. An overall score was calculated (0–100) by using the following equation: (25 × (sum of answered question scores/number of questions answered)). Participants were also asked to grade five symptoms associated with the CVS. The symptoms of fatigue, burning, dryness, blurred vision, and dullness of vision was ranked on a scale of 0 to 6 (0 = “no symptom”, 6 = “very severe symptom”) and the average aggregate score for the two test groups are reported.

### Statistical analysis

The Statistical package for the Social Sciences software, version 18.0 (SPSS, Inc., Chicago, IL) was used for analysis. Data are shown as means ± SEM. The normality of distribution for all variables was verified by the Kolmogorov-Smirnov test. The Student *t* test for normally distributed variables or Mann-Whitney U test for non-normally distributed variables was used to compare continuous variables between two groups. One-way analysis of variance followed by a Tukey-Kramer post hoc test for comparison of three groups. Categorical variables were compared with the chi square test. To compare changes in the various parameters in each time point, repeated measures analysis of variance, followed by Bonferroni’s post-hoc tests, was used. The assumption of sphericity was verified using the Mauchly’s test. If the data were violated, the Epsilon Greenhouse–Geisser correction was applied. A *P* < 0.05 (*) was considered statistically significant and *P* < 0.01 (**) as highly significant.

## Results

### C3 and dapa analogues on TRPM8 activity in vitro

**Fig. 2:**
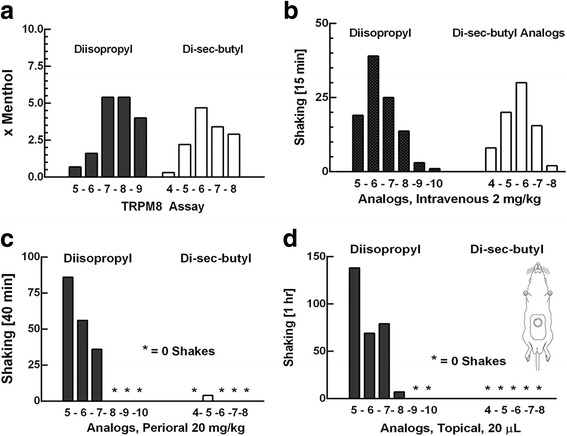
Pharmacological activities of 1-dialkylphosphorylalkanes (Dapa). (**a**) Relative potencies of Dapa to menthol in TRPM8 assay. The number on the abscissa represent the *n*-alkyl side-chain of 4–5–6-7-8-9-10 carbons, corresponding to a butyl, pentyl, hexyl, heptyl, octyl, nonyl and decyl group, respectively. (**b, c, d**) Shaking frequency of anesthetized rats (*n* = 3 to 6 per group) is counted for 15 min, 40 min, and 1 h after intravenous 2 mg/kg, perioral 20 mg/kg, or topical 20 μL application of Dapa. From this pattern of response, C3 (the nonyl analogue) is selected as the lead candidate. The term x Menthol on the ordinate of Fig. 2a is potency relative to I-menthol, wherein menthol = 1

**Fig. 3:**
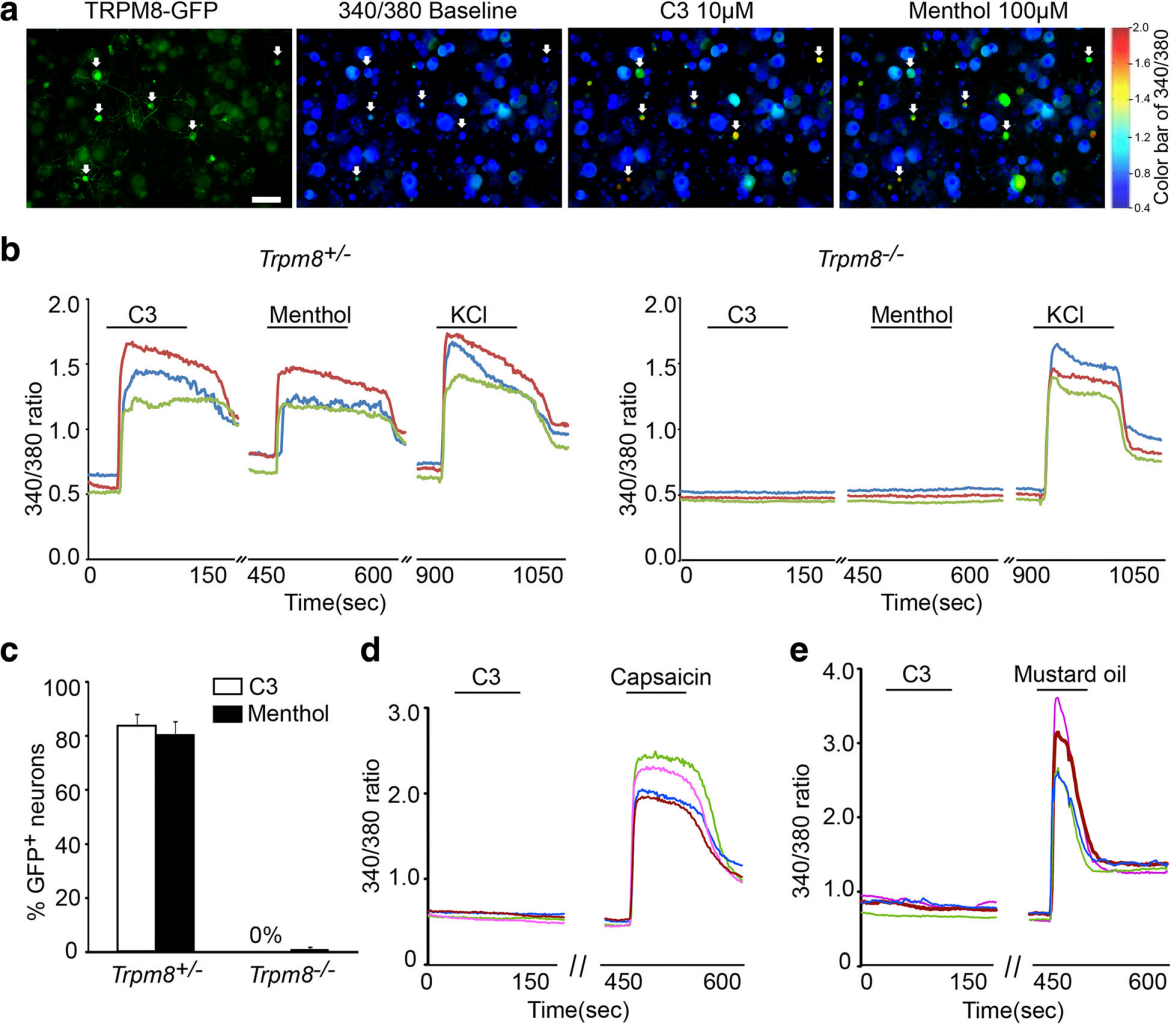
C3 is a specific agonist of TRPM8. (**a**) Image shows fluorescence emission of Ca^2^+ influx in response to excitation by Fura2-AM loading. C3 (10 μM) activates neurons (green) from cultured*Trpm8* ^*EGFPf/+*^transgenic mice which are also menthol (100 μM) sensitive. White arrows indicate the responsive neurons. Scale bar: 100 μm. (**b**) Representative calcium traces for C3 and menthol on *Trpm8* ^*EGFPf/+*^ (*Trpm8* ^*+/−*^) or*Trpm8* ^*EGFPf/EGFPf*^ (*Trpm8* ^*−/−*^) transgenic neurons. High concentration of K^+^ (KCl) was used to identify the total number of neurons. (**c**) Quantification of C3 and menthol activation on *Trpm8* ^*+/−*^(*n* = 104) and*Trpm8* ^*−/−*^transgenic neurons (*n* = 136). High concentration of K^+^ (KCl) was used to identify the total number of neurons. (**d**, **e**) C3 does not activate mouse TRPV1 or human TRPA1 ion channels in heterologous KNRK cells, which are activated by positive control substances, TRPV1 capsaicin (10 μM), or TRPA1 mustard oil (100 μM), respectively)

A number of Dapa analogues with 11 to 16 total carbons were synthesized and assayed on CHO cells transfected with the hTRM8 plasmid and calcium entry into cells was used as an indicator of bioactivity. The diisopropylphorylalkanes and di-sec-butylalkanes with 13 to 15 total carbons were several times more potent than menthol and had the lowest half effective concentration (EC_50_) (Fig. 2a, Additional file 1: Table S1). C3, with an EC_**50**_ of 0.9 μM compared to a menthol value of 3.6 μM, was selected for further analysis. In primary sensory neurons isolated and cultured from *TRPM8* ^*EGFP/+*^ mice, in which GFP is expressed in TRPM8^+^ neurons, C3 activates calcium entry but not in TRPM8-deficient sensory neurons from *TRPM8* ^*−/−*^ mice (Fig. 3a-c). C3 was inactive at 10 μMin cells transfected with the TRPV1 and TRPA1 plasmids (Fig. 3d, e), but the positive controls, capsaicin and mustard oil was active. These results show that C3 is a selective and specific agonist of TRPM8 on sensory neurons.

### C3 activity in laboratory animals

The Dapa analogues were administered to pentobarbital-anesthetized rats by intravenous, perioral, and topical routes and the number of “wet dog shakes (WDS)” were counted as an endpoint of activity (Fig. 2b–d). WDS are rapid, vigorous, rotational movements of the animal about its spinal axis and is indicative of sensory stimulation (Additional file 2: Movie S1) [49, 50]. The diisopropyl analogues were more active than the di-sec-butyl analogues by the perioral and topical route, indicating easier passage of such analogues past a keratinized barrier. C3 was the optimal diisopropyl analogue because it had a low TRPM8 EC_**50**_, was not active by the oral route, but active when given intravenously. The nonane group was likely to permit a longer duration of action than the hexyl, heptyl, or octyl equivalent because of increased hydrophobocity. The di-sec-butyl analogues were not active by topical or oral administration, indicating greater lipophilic characteristics. These analogues were active by intravenous injection (Fig. 2b).

**Fig. 4:**
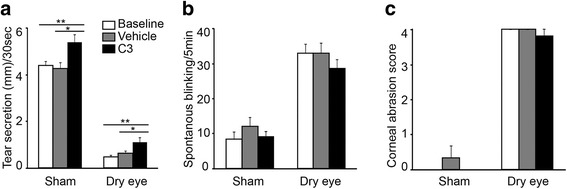
Tear secretion in dry eye mice is increased via application of C3 on upper eyelid. (**a**) Tear secretion is increased via topical application of C3 compared to the vehicle (saline), both in sham group (5.4 ± 0.5 vs. 3.8 ± 0.5, *n* = 5) and dry eye mice (1.3 ± 0.4 vs. 0.5 ± 0.1, *n* = 6). (**b, c**) Spontaneous blinking and corneal abrasion are not affected by vehicle or C3 application in sham group or dry eye mouse model. All data are presented as means ± SEM. Statistical significances were calculated using t-test. *P < 0.05. **P < 0.01

To determine if C3 affected tear secretion, we tested its effect in mice. Mice were held firmly and 2 μL of C3 or vehicle was wiped gently on eyelid by pipette from nasal side to bitemporal. Tear secretion was increased by C3 in sham-operated animals and in mice in which the extraorbital lacrimal glands were surgically removed (Fig. 4a) [51]. In this model of dry eye disease, eye blinking frequency is also increased, as well as the grades of corneal abrasions, but these indicators of DED were not affected by the single administration of C3 (Fig. 4b,c). The positive results in an animal model of DED justified further studies in human subjects.

### Ocular margin delivery of a TRPM8 agonist

Eye drops represent ~90% of formulations used for treatment of anterior eye disorders [30, 52–54]. We therefore tested C3, 0.1 mg/mL in water, instilled as a 50 μL eye drop to the lower conjunctival fornix of volunteers, with vehicle controls (*n* = 5 per group). Subjects were asked to rate discomfort, blurring, and coolness sensations, on a scale of 0 (no effect) to 5 (intense effect) at 5, 10, and 15 min after instillation. C3 did not cause significant blurring at these tested intervals, however, there was discomfort at the 5 min test period (score 1.6 vs 0.2), but not at 10 or 15 min. Cooling sensation scores were significant at 5 min (2.6 vs 0.2), but there were not much cooling at 10 (0.8 vs 0.2) or 15 min (0.2 vs 0.2). It appeared as if eye drops are a relatively inefficient method of C3 delivery because the drop splashes in a confined space and the time for the active ingredient to be retained on target receptors is short. Furthermore, the initial period of discomfort is not desirable. To determine if there were other TRPM8 targets on the orbit, we examined the mouse eye with a selective TRPM8-GFP reporter line.

**Fig. 5:**
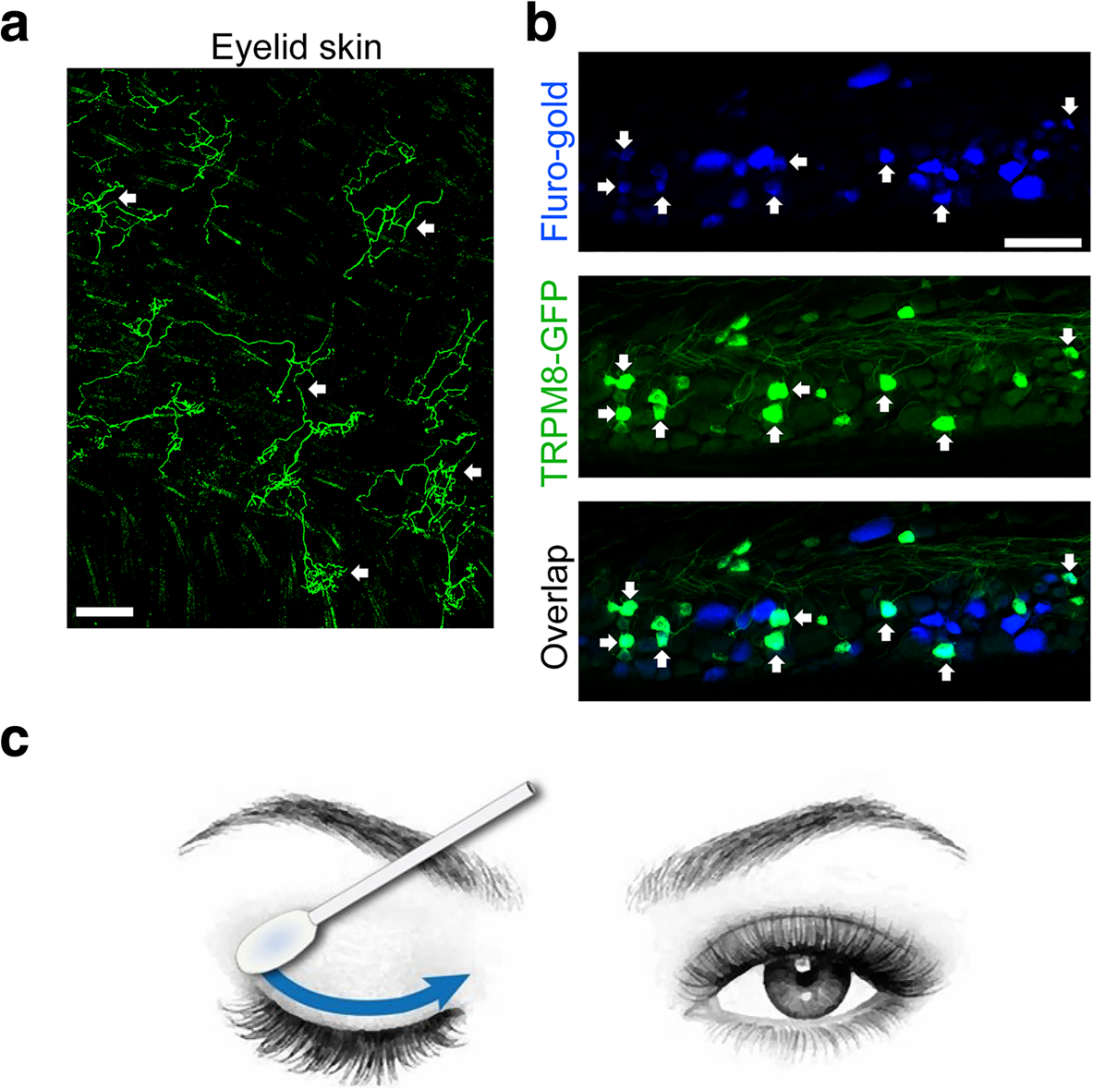
Upper eyelid is densely innervated by TRPM8 fibers and is the target of drug delivery. (**a**) TRPM8-expressing sensory fibers (green) densely innervate the skin of upper eyelid as revealed by the whole-mount staining of TRPM8-GFP from*Trpm8* ^*EGFPf/+*^transgenic mice. White arrows indicate *Trpm8* ^*EGFPf/+*^fiber axons and terminals. (**b**) Trigeminal ganglion neurons which innervate the upper eyelid are revealed by microinjection of neuronal retrograde tracer, Fluoro-Gold™, in the upper eyelid. White arrows indicate TRPM8^+^/fluorogold^+^ neurons. Scale bars in (**a)**: 250 μm, (**b)**: 100 μm. (**c**) The method to topically apply test solutions to target TRPM8 on the eyelid margins

Similar to other reports, we found TRPM8 immunoreactivity on the rodent cornea [15]. Unexpectedly, a dense TRPM8 innervation was also revealed in the eyelid margins, especially at the base of the eyelash hair shafts (Fig. 5a), but hardly any TRPM8 fibers were found on the conjunctiva (Additional file 1: Figure S1). The sensory fibers innervating the upper eyelid and cornea are located in the V1 ophthalmic branch of the trigeminal nerve (Additional file 1: Figure S2). We speculated that TRPM8 signals from the ocular margins may be perceived as cooling signals for the entire eye surface and gate nociceptive input from the cornea. Delivery of C3 to the ocular margins may also decrease the initial discomfort by reducing direct contact of the drug with corneal nociceptors that irritate and cause pain. To apply C3 to the ocular margins, we first used C3 on a gauze wipe, then on a cotton-tipped applicator (Fig. 5c). The C3 was wiped onto the upper eyelids, touching the lashes, in a lateral to a medial direction; thus mimicking the natural direction of tear secretion. One would expect the eyelash shaft to serves as a wick to distribute the aqueous solution across the eye margin and merge with the precorneal film which is also aqueous. The keratinized epithelial cushion, called the eyelid wiper [44], would then push the solution across the ocular surface. Surprisingly, the wipe method for C3 allowed comfortable and prolonged ocular surface cooling to be achieved without discomfort.

To ascertain if this method of drug delivery allowed even distribution across the ocular surface, we compared the wipe method to eye drops using a 1% fluorescein solution as marker, and examined the margins and cornea with a slit lamp. It was estimated that wiping distributed less than 5% of the fluorescein over the cornea compared with conventional eyedrops. The wipe method of delivery is not a conventional method of drug delivery to the ocular surface, but appears to have merit for drugs that may irritate by acting on corneal nociceptors [55]. In practice, subjects using the wipe method commented that it was more convenient and easier to use than conventional eye drops.

### C3 effects on subjects with ocular discomfort

The baseline characteristics of the enrolled subjects in the two randomized groups and the designs of the studies are shown elsewhere (Additional file 1: Tables S2, S3 and Figs. S3, S4). Sixty subjects (*n* = 30 per group) participated in the single dose study, and forty subjects (*n* = 20 per group) completed the 2 week study.

**Fig. 6:**
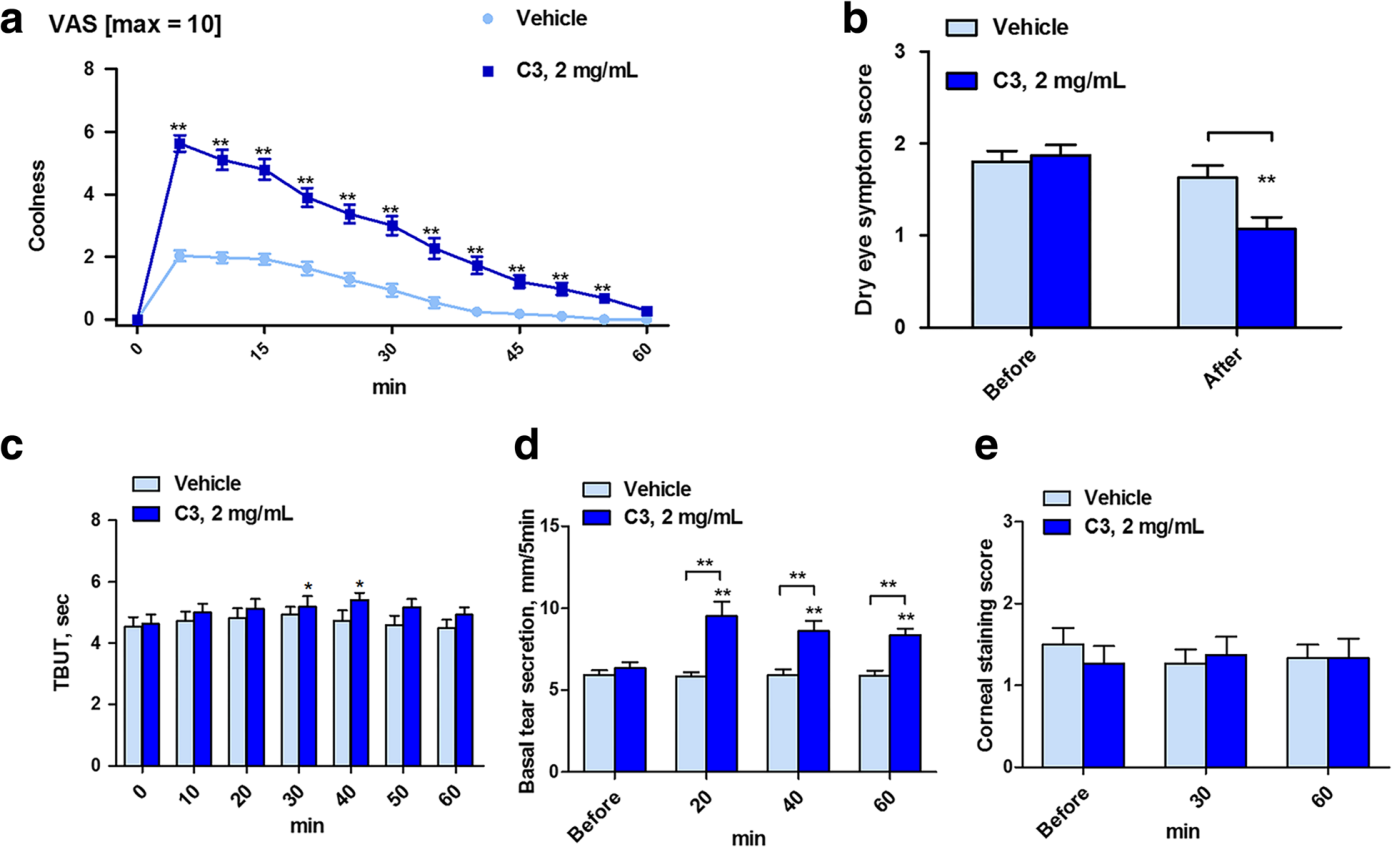
Sensation of coolness and changes in ocular parameters after a single application of vehicle or C3, 2 mg/mL. (**a**) Visual analogue scale (VAS) for coolness on ocular surface. (**b**) Dry eye symptom score. (**c**) Tear break-up time (TBUT) in sec. (**d**) Basal tear secretion in mm. (**e**) Keratoepitheliopathy score. * *P* < 0.05, **P < 0.01, compared to baseline value and vehicle (*n* = 30 in each group)

C3 increased the VAS for ocular cooling score within 5 min after application, and lasted for an average of 46 ± 2 min (Fig. 6a). The C3 treatment scored higher than the vehicle at every time point and there was a clear-cut pharmacological effect. The sensations reported were of refreshing and dynramic cooling., with an energizing effect. None of patients reported sensing ocular pain or irritation after topical application of vehicle or C3. The dry eye symptom scores improved significantly after C3 compared to baselines and was not seen with the vehicle controls (Fig. 6b). The TBUT was significantly elevated above baseline at 30 min and 40 min after C3 (Fig. 6c). The intergroup comparison of the TBUT did not show a significant difference. The basal tear secretion significantly increased at 20 min, 40 min, and 60 min after C3, but not in the vehicle control group (Fig. 6d). The intergroup comparison was highly significant for the three time points tested. No differences were observed in KEP scores of the two treatment groups (Fig. 6e).

**Fig. 7:**
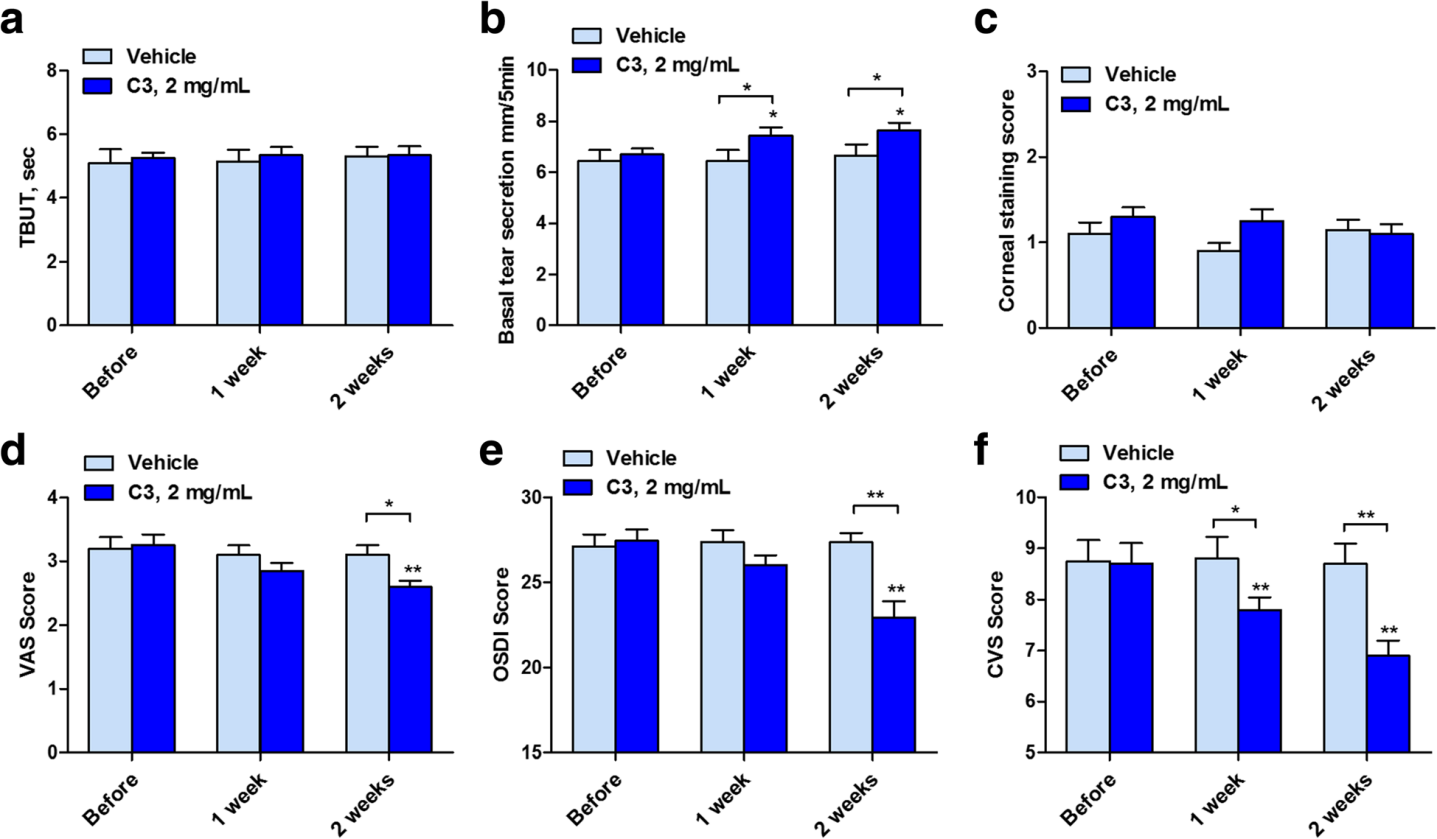
Changes in ocular parameters after four times a day application of vehicle or C3, 2 mg/mL for 2-weeks. (**a**) Tear break-up time (TBUT) in sec. (**b**) Basal tear secretion in mm. (**c**) Keratoepitheliopathy score. (**d**) Visual analogue scale (VAS) for ocular discomfort. (**e**) Ocular surface disease index (OSDI) score. (**f**) Computer vision syndrome (CVS) symptom score. * *P* < 0.05, **P < 0.01, compared to baseline value and vehicle (*n* = 20 in each group)

The total of 40 patients (*n* = 20 per group) who completed the 2-week course of study did not differ in age, sex, and baseline symptoms, or in ocular surface parameters and were young adults with a mild degree of DED. The weight of the applicators used in this study decreased by 80 ± 5 mg (week 1) and 83 ± 4 mg (week 2) for the vehicle group and 77 ± 7 mg (week 1) and 81 ± 4 mg (week 2) for the C3 group, respectively. As the recommended usage was for four times a day the average volume per application off-loaded each time the applicator was used is about 20 μL of water or 40 μg of C3 for both eyes for a 2 mg/mL solution. The changes in ocular parameters after vehicle or C3 showed no significant changes in TBUT and KEP at week 1 or week 2 (Fig. 7a, c). The basal tear secretion was significantly increased in the C3 group at 1 and 2 weeks when compared to baseline and when compared to the vehicle (Fig. 7b). The changes in ocular symptoms scores, assessed by three questionnaires, showed that the severity of symptoms assessed by VAS score or by the total OSDI score were significantly improved at week 2 but not at week 1 after C3 when compared with the vehicle (Fig. 7d, e). The CVS type of symptoms was significantly improved at both 1 and 2 weeks after C3 (Fig. 7f). No significant adverse effect such as ocular pain, irritation, or discomfort was reported from both groups during the 2 weeks study.

## Discussion

The cooling property of the dialkylphosphorylalkanes on the tongue of test subjects was first reported in 1978 [33], but only one observation on this topic, on the TRPM8 EC_**50**_ of 1-di-sec-butylphosphorylheptane, was published thereafter [56]. These molecules are attractive for ocular applications because they dissolve in aqueous media at concentrations, e.g. 0.5 to 5 mg/mL, which provide refreshing sensations of heat abstraction. C3 was selected as the lead candidate because it was selective for TRPM8, had minimal irritation, and an optimal duration of drug action.

TRP cation channels are coupled to sensory stimuli such as chemical irritants and temperature change [17, 57–61]. In the TRP family, TRPM8 is categorized as a cold-sensitive receptor, with a threshold of ~25 °C and is rapidly activated by a drop in temperature. TRPM8 immunoreactivity is present at the sensory nerve ending of the ophthalmic branch of the trigeminal nerve. TRPM8 in vitro responds to temperature reduction, and is activated by chemicals such as menthol and icilin. Cold-sensitive ocular units, like skin receptors, exhibit spontaneous activity; and it is speculated that TRPM8 regulates lacrimal function because it responds to evaporative cooling and hyperosmolar stimuli, and because TRPM8 knock-out mice have a reduced rate of basal tear secretion.

In the current study, topical administration of TRPM8 agonist C3 to the closed eyelids quickly led subjects to feel coolness on the ocular surface, and the sensations remained on average for 46 min. After a single application of C3 or daily application for 2 weeks, symptoms of dry eye were improved and basal tear secretion was increased. No subjects in our study complained of ocular irritation or pain. The change in basal tear secretion was clearly significant and of a magnitude that may affect dry eye discomfort.

**Fig. 8:**
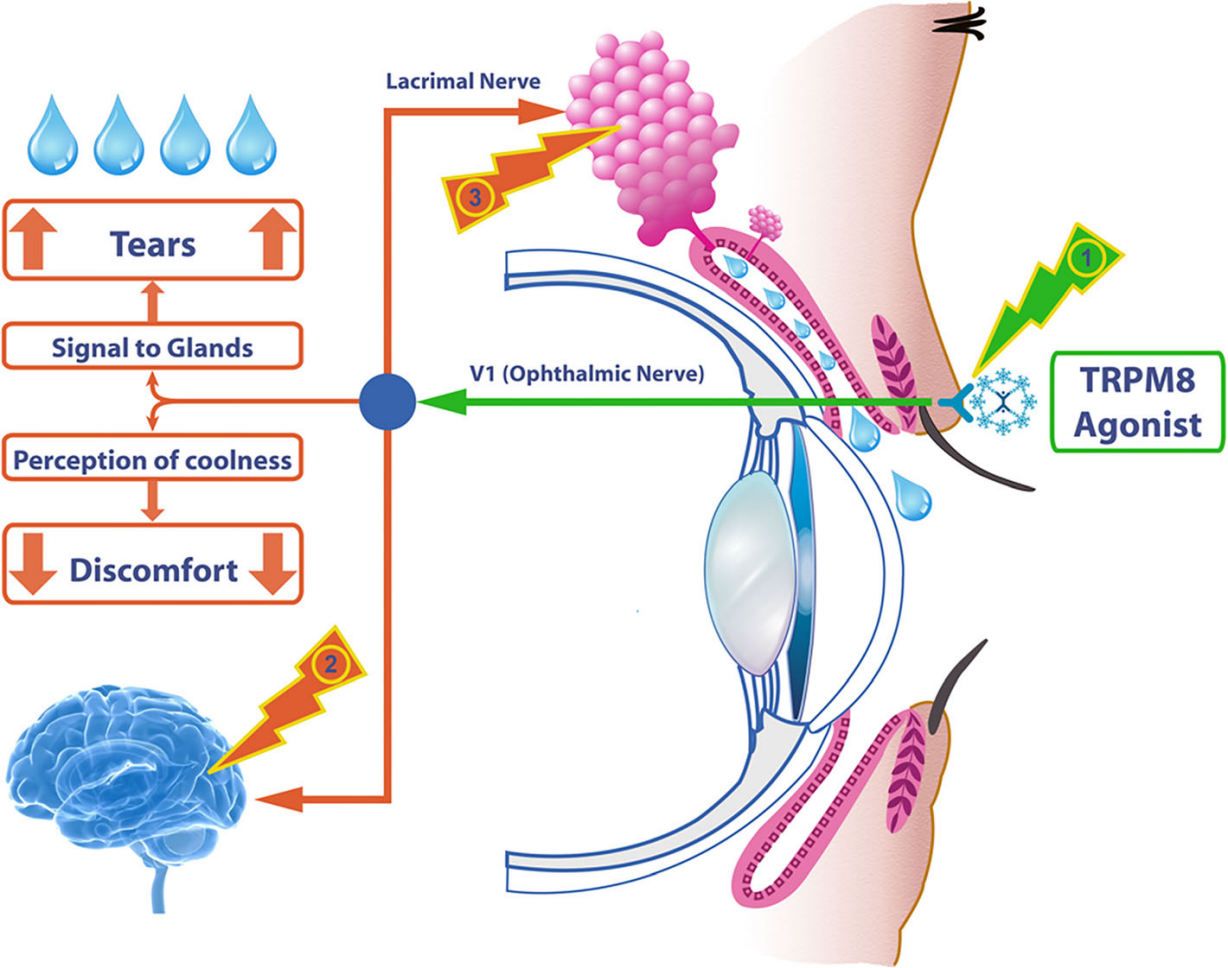
Mechanisms of action of C3 in the relief of dry eye symptoms. C3 applied over the upper eyelid stimulates TRPM8 located in the eyelid margin (1) and this signal is passed via the ophthalmic nerve (V1) towards the central nervous system. The perception of coolness reduces ocular discomfort (2) and efferent signals for increased tear secretion may also be activated (3)

The mechanisms underlying the C3 action are not precisely understood. In previous studies with a related analogue, 1-di-sec-butylphosphorylpentane, corneal sensitivity measured by Cochet-Bonnet esthesiometry, was not affected by a 2 mg/mL solution sprayed onto the closed eyelid [62]. This analogue was also evaluated at 5 μM in hNav1.7 (sodium channels) and did not have lidocaine-like activity [63]. Hence, the effect of C3 on the ocular surface is most likely not a local anesthetic action. The method of drug delivery used here, that of wiping ~10 μL per eye or less of solution to the ocular margins, may be important for avoiding excess stimulation of polymodal neurons that mediates nociceptive sensations in ocular surface [11, 64]. It is noted that delivery of a 0.1 mg/mL solution of C3 as an eye drop in a larger volume of 70 μL elicits eye discomfort, without cooling lasting longer than 10 min. This is 5% of the 2 mg/mL concentration applied via wiping in Expt. 2. Minimizing contact with corneal nociceptors linked to sting, irritation, and pain, may be a key factor to the success of the wiping method of drug delivery. A summary illustration of the mechanisms of action of C3 in the relief of dry eye discomfort is shown in Fig. 8.

In DED there is a deficiency of tear quality or tear secretion accompanied by sensations of dryness. In our study, TBUT was transiently increased by C3 at 30 to 40 min after application, an effect that could be secondary to the increased tear secretion seen at the same time [40, 65, 66]. No changes were observed in TBUT in the 2-week study. The increase in tear secretion, as measured by the Schirmer score, was an objective sign of C3 effect and may be due to stimulation of corneal, conjunctival, or lacrimal gland receptors [55, 60, 67, 68]. Since the data were collected only 60 min after single application of agents, and 2 weeks of daily application, we do not know if longer application can lead to a better outcome. In addition, because the mean baseline corneal epithelial damage was mild and TBUT was not so low in our study, dramatic improvement might be hard to be expected. The mechanism underlying this response and its optimal posology requires further investigation with longitudinal studies involving longer applications of TRPM8 agonist in patients with more severe ocular surface damage. The relief of symptoms was most likely from the cooling effect of C3. Although our study was designed to be double-blinded, strict masking would have been difficult. It should be cautioned that the sensory effects of C3 were quickly detected by the test subject and was distinguished from vehicle, so even though a double-masked design was used, bias may be present in the response to the questionnaire.

It is interesting to note that there were no overt adverse effects such as ‘skin irritation’ and ‘potential corneal effects.’ This lack of adverse effects is most likely due to the water-solubility of the molecule, which is less likely to accumulate in skin or body fluids. As our study includes preliminary data of short-term effect of C3, longer follow up are required to determine if there are potential corneal effects.

The average age of the subjects in the first experiment (single dose study) was 29 years and in the second experiment (2 weeks study) was 23 years. As we did not include MGD patients in the study, the C3 treatment may not be as effective in a subgroup of patients with evaporative type DED. Further investigations including the patients with older group of evaporative type DED are necessary to clarify this issue. The baseline corneal epithelial damage and symptomatology studied here were mild, and dramatic improvement may not be easy to detect. Studies of C3 in patients with more severe ocular surface damage should reveal the range of C3 drug efficacy.

## Conclusion

In summary, we have created and tested a novel water-soluble TRPM8 agonist on the human ocular surface. The preliminary results indicate that this treatment facilitates basal tear production in patients with mild to moderate DED and provides a cooling sensation for symptom relief. C3 is a promising candidate for further study of dry eye disease.

## Additional files

Additional file 1: Figures S1–S3 and Tables S1–S3. Figure S1. Cornea, but not conjunctiva, is highly innervated by TRPM8 sensory fibers. **Figure S2.** The cell bodies of sensory fibers innervating the upper eyelid and cornea are located in the V1 ophthalmic branch of the trigeminal ganglion. **Figure S3.** Flow diagram for the design of Expt 1. **Figure S4.** Flow diagram for the design for Expt 2. **Table S1.** EC_50_ and relative potency of compounds on TRPM8. **Table S2.** Baseline characteristics of the enrolled subjects in Expt 1. **Table S3.** Baseline characteristics of the enrolled subjects in Expt 2. (DOCX 4087 kb)

Additional file 2: Movie S1. Shaking response to a 1-diisopropylphosphorylnonane. Video recording 20 to 40 min period after oral dosing with C3 at 50 mg/kg. C3 was dissolved in 5% ethanol-95% R-1,2-propanediol. The total observation period was 120 min. The total number of shaking in the most active rat was 56 shakes, which only occurred in this interval. In other experiments, no body shakes was observed after oral dosing with 20 mg/kg in both the anesthetized and unanesthetized state. (MP4 3699 kb)

## Abbreviations

C3: Cryosim-3
CHO: Chinese Hamster Ovary
CVS: Computer vision syndrome
Dapa: 1-dialkylphosphorylalkane
DED: Dry eye disease
EC50: Half effective concentration
GS-MS: Gas chromatography-mass spectrometry
HBPS: HEPES-buffered physiological saline
KEP: Keratoepitheliopathy
KNRK: Kirsten murine sarcoma virus transformed kidney
OSDI: Ocular surface disease index
TBUT: Tear break up time
THF: Tetrahydrofuran
TIC: Total ion chromatogram
TRP: Transient receptor potential
TRPM8: Transient receptor potential melastatin 8
TRPV1: Transient receptor potential vanilloid 1
VAS: Visual analogue scale
WDS: Wet doc shakes
