# Supplementary material for: A novel TRPM8 agonist relieves dry eye discomfort
Source: BMC Ophthalmol. 2017 Jun 26;17:101. doi: 10.1186/s12886-017-0495-2 (PMC5485613; doi:10.1186/s12886-017-0495-2)
Supplement: Supplementary file 1 — Cornea, but not conjunctiva, is highly innervated by TRPM8 sensory fibers. Figure S2. The cell bodies of sensory fibers innervating the upper eyelid and cornea are located in the V1 ophthalmic branch of the trigeminal ganglion. Figure S3. Flow diagram for the design of Expt 1. Figure S4. Flow diagram for the design for Expt 2. Table S1. EC50 and relative potency of compounds on TRPM8. Table S2. Baseline characteristics of the enrolled subjects in Expt 1. Table S3. Baseline characteristics of the enrolled subjects in Expt 2. (DOCX 4087 kb) [file 12886_2017_495_MOESM1_ESM.docx]

**Additional file 1.**


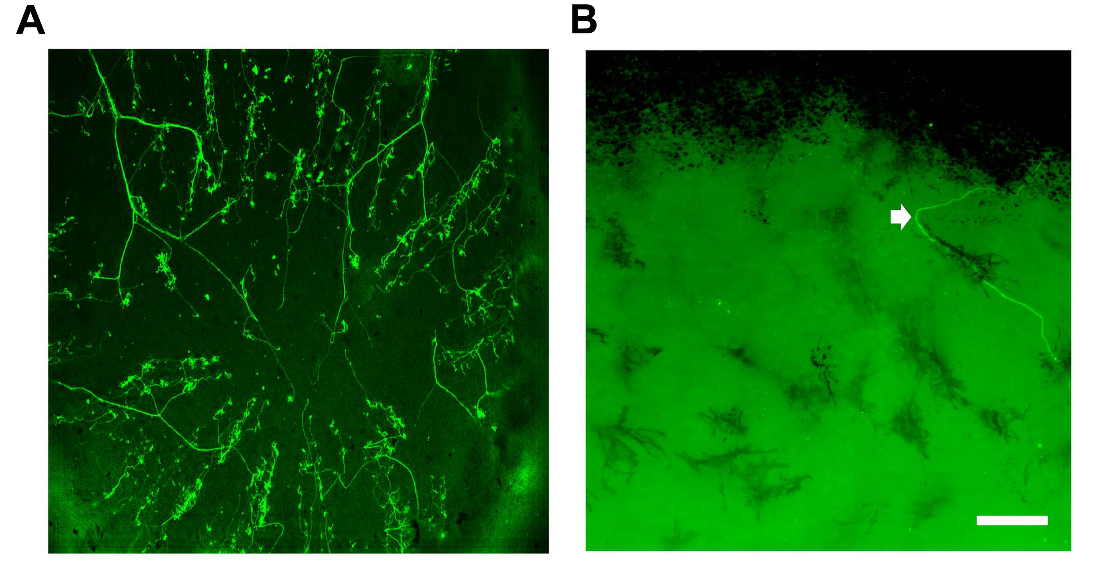


**Fig. S1. Cornea, but not conjunctiva, is highly innervated by TRPM8 sensory fibers.** (A) TRPM8-expressing sensory fibers (green) densely innervate the cornea from *Trpm8^EGFPf/+^* transgenic mice. (B) Conjunctiva is seldom innervated by TRPM8^+^ fibers. White arrows indicate *Trpm8^EGFPf/+^* fiber axons go through the conjunctiva under the mucosa layer. Scale bar: 250μm.


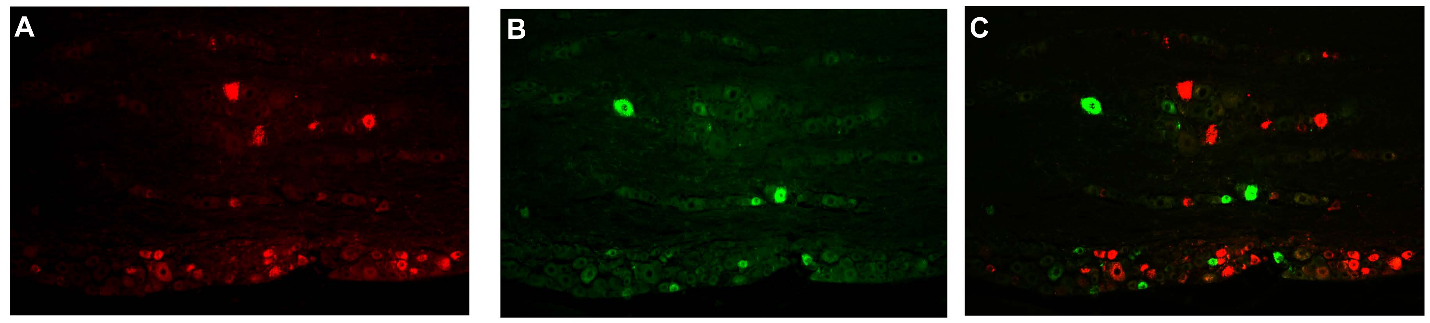


**Fig. S2. The cell bodies of sensory fibers innervating the upper eyelid and cornea are located in the V1 ophthalmic branch of the trigeminal ganglion.** (**A**) WGA-Alexa Fluor® 555 labeled trigeminal neurons for upper eyelid. (**B**) WGA-Alexa Fluor® 488 labeled trigeminal neurons for the cornea. (**C**) Merge of the fluorescence in the same trigeminal ganglion section. Scale bar: 100μm.

**
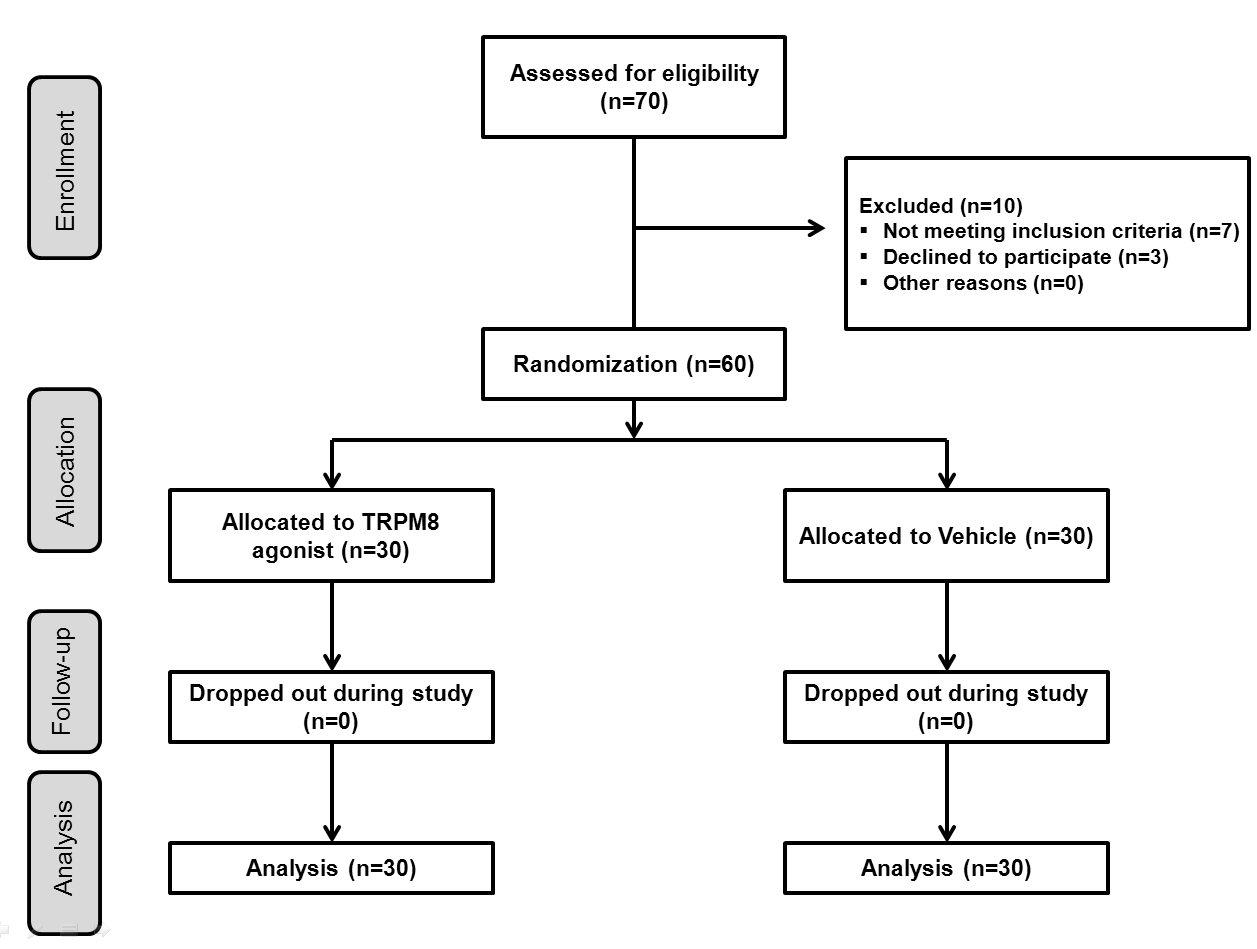
Fig. S3. Flow diagram for the design of Expt 1.** Subjects received a single application of C3, 2 mg/mL, wiped across the upper eyelid with a gauze pad.

**
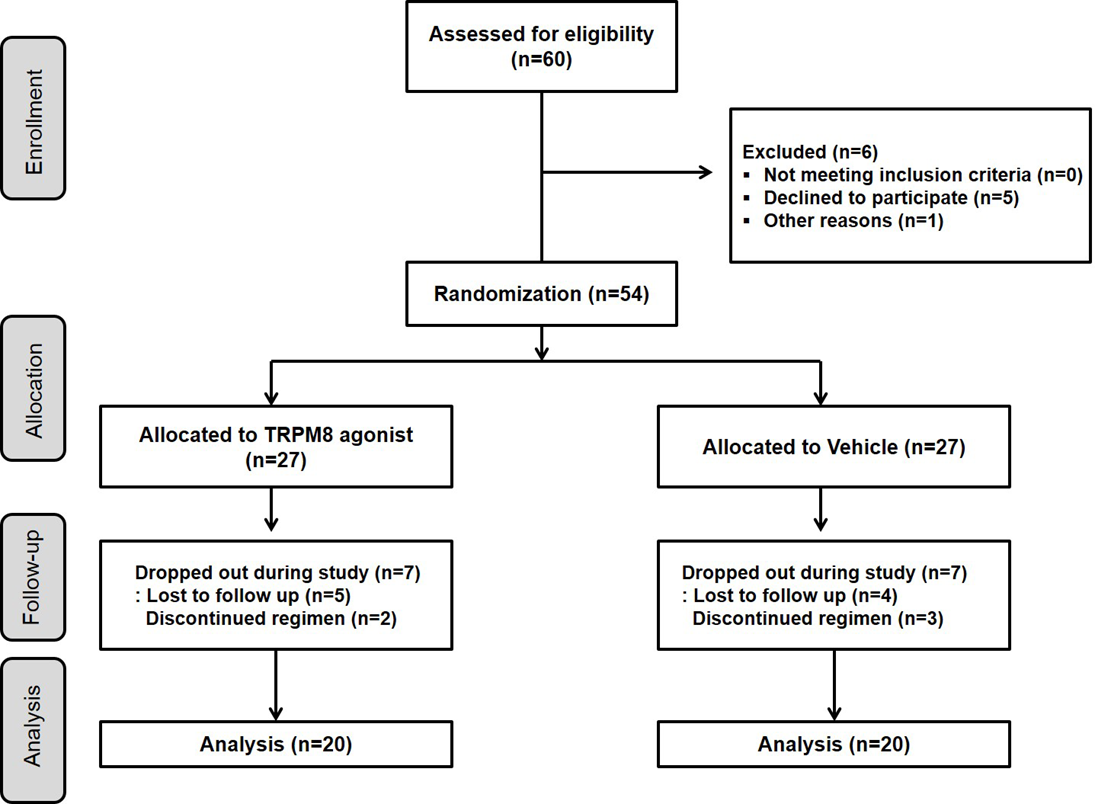
Fig. S4. Flow diagram for the design for Expt 2.** Subjects received 14 applicators containing 1 mL of C3, 2 mg/mL, and instructions to wipe the applicator across the upper eyelid four times a day at 6 hr intervals.

**Table S1.** **EC_50_ and relative potency of compounds on TRPM8.** The EC**_50_** of the more potent compounds, 1-diisopropylphosphoryl-C**_7_** to C**_9_** and 1-di-sec-butylphosphoryl-C**_5_** to C**_8_** fell within a narrow range with overlapping 95% Confidence Intervals. By contrast, structural modifications to the diisobutylphosphoryl moiety or branching of the alkane group to 3-methyl-pentane resulted in a significant loss of activity.

| **Chemical** | **EC_50_ µM** | **95% CL** | **I-Menthol** |
| --- | --- | --- | --- |
|  |  |  |  |
| l-menthol | 3.8 | 2.5 to 5.6 | 1.0 |
| 1-diisopropylphosphoryl-pentane | 5.6 | 4.4 to 7.2 | 0.7 |
| 1-diisopropylphosphoryl-hexane | 2.4 | 1.5 to 4.0 | 1.6 |
| 1-diisopropylphosphoryl-heptane | 0.7 | 0.5 to 1.0 | 5.4 |
| 1-diisopropylphosphoryl-octane | 0.7 | 0.5 to 1.0 | 5.4 |
| 1-diisopropylphosphoryl-nonane | 0.9 | 0.4 to 2.5 | 4.0 |
| 1-di-sec-butylphosphoryl-4 | 14.5 | 7 to 29 | 0.3 |
| 1-di-sec-butylphosphoryl-pentane | 1.7 | 1.0 to 2.9 | 2.2 |
| 1-di-sec-butylphosphoryl-hexane | 0.8 | 0.5 to 1.3 | 4.7 |
| 1-di-sec-butylphosphoryl-heptane | 1.1 | 0.6 to 2.3 | 3.4 |
| 1-di-sec-butylphosphoryl-octane | 1.3 | 0.7 to 2.3 | 2.9 |
| 1-diisobutylphosphoryl-pentane | 24 | 8 to 76 | 0.2 |
| 1-di-sec-butylphosphoryl-3-methyl-butane | 4.2 | 1.6 to 10.8 | 0.9 |

**Table S2. Baseline characteristics of the enrolled subjects in Expt 1**

|  | **Vehicle** | **C3, 2 mg/mL** | ***P* value** |
| --- | --- | --- | --- |
| Age (years) | 28.57 ± 1.23 | 29.70 ± 4.47 | 0.446^a^ |
| Gender (men/women) | 4/26 | 5/25 | 0.718 ^b^ |
| Symptom score (0-4) | 1.80 ± 0.12 | 1.87 ± 0.11 | 0.691 ^a^ |
| TBUT (seconds) | 4.53 ± 0.26 | 4.63 ± 0.26 | 0.784^a^ |
| Schirmer score (mm/5min) | 5.93 ± 0.24 | 6.33 ± 0.32 | 0.317^a^ |
| Keratoepitheliopathy score | 1.50 ± 0.16 | 1.27 ± 0.17 | 0.331^a^ |
| Corneal sensitivity (mm) | 60.00 ± 0.00 | 59.33 ± 0.46 | 0.155 ^a^ |

C3 = cryosim 3, TBUT = tear break-up time

Data are expressed as the mean ± SEM.

^a^Student-*t* test.

^b^Chi-square test.

**Table S3. Baseline characteristics of the enrolled subjects in Expt 2**

|  | **Vehicle** | **C3, 2 mg/mL** | ***P* value** |
| --- | --- | --- | --- |
| Age (years) | 22.25 ± 0.56 | 23.75 ± 0.83 | 0.478 ^a^ |
| Gender (men/women) | 6/14 | 8/12 | 0.741 ^b^ |
| OSDI (0-100) | 27.12 ± 0.72 | 27.45 ± 0.67 | 0.495 ^a^ |
| VAS (0-10) | 3.20 ± 0.18 | 3.25 ± 0.18 | 0.841 ^a^ |
| Computer vision syndrome score (0-30) | 8.75 ± 0.41 | 8.70 ± 0.40 | 0.925 ^a^ |
| TBUT (seconds) | 5.25 ± 0.18 | 5.10 ± 0.43 | 0.883 ^a^ |
| Schirmer score (mm/5min) | 6.70 ± 0.24 | 6.65 ± 0.29 | 0.718 ^a^ |
| Keratoepitheliopathy score | 1.10 ± 0.13 | 1.30 ± 0.11 | 0.242 ^a^ |
| Corneal sensitivity (mm) | 59.50 ± 0.50 | 59.00 ± 0.69 | 0.799 ^a^ |

C3 = cryosim 3, OSDI = ocular surface disease index, TBUT = tear break-up time, VAS = visual analogue scale

Data are expressed as the mean ± standard error of mean.

^a^Mann-Whitney U test.

^b^Chi-square test.
